# In Wrong Anticipation - Miscalibrated Beliefs between Germans, Israelis, and Palestinians

**DOI:** 10.1371/journal.pone.0156998

**Published:** 2016-06-16

**Authors:** Sebastian J. Goerg, Heike Hennig-Schmidt, Gari Walkowitz, Eyal Winter

**Affiliations:** 1 Department of Economics, Florida State University, Tallahassee, Florida, United States of America; 2 Max Planck Institute for Research on Collective Goods, Bonn, Germany; 3 Laboratory for Experimental Economics, University of Bonn, Bonn, Germany; 4 Department of Health Management and Health Economics, University of Oslo, Oslo, Norway; 5 Corporate Development and Business Ethics, University of Cologne, Cologne, Germany; 6 The Center for the Study of Rationality, The Hebrew University of Jerusalem, Jerusalem, Israel; George Mason University, UNITED STATES

## Abstract

The reconcilability of actions and beliefs in inter-country relationships, either in business or politics, is of vital importance as incorrect beliefs on foreigners’ behavior can have serious implications. We study a typical inter-country interaction by means of a controlled laboratory investment game experiment in Germany, Israel and Palestine involving 400 student participants in total. An investor has to take a risky decision in a foreign country that involves transferring money to an investee/allocator. We found a notable constellation of calibrated and un-calibrated beliefs. Within each country, transfer standards exist, which investees correctly anticipate within their country. However, across countries these standards differ. By attributing the standard of their own environment to the other countries investees are remarkably bad in predicting foreign investors’ behavior. The tendency to ignore this potential difference can be a source of misinterpreting motives in cross-country interaction. Foreigners might perceive behavior as unfavorable or favorable differentiation, even though—unknown to them—investors actually treat fellow-country people and foreigners alike.

## Introduction

The reconcilability of actions and beliefs in inter-country relationships is of vital importance as incorrect beliefs on foreigners’ behavior can have serious implications. Tim Clissold, a Western businessman, for example, concluded after his devastating experiences in China: “[B]illions were lost by foreign investors. [..] I had been forced to dismantle my assumptions about China and relearn all the basics, but many investors still appeared supremely confident that China would eventually view the world their way” pp. 298/9 in [1]. While the failing of joint ventures due to the misalignment of partners’ expectations and behavior can result in high economic costs, these negative impacts are less dramatic in comparison to the possible effects on international relations. It has, for instance, been conjectured that the 1973 Yom Kippur War could have been prevented if the Nixon administration had believed Egyptian President Sadat’s announcements of going to war in case the United States would not actively engage in reconciliation negotiations between Egypt and Israel [2],[3]. These examples highlight fatal ramifications resulting from incorrect predictions of foreigners’ behavior. Consequences would have been less fatal had the protagonists realized that such misalignment existed and, in addition, had they well understood the causes for and the mechanisms behind the (mis)calibration. In this paper, we direct our attention to this phenomenon that has largely been neglected so far.

If misguided beliefs in interactions with foreigners are a regular phenomenon, it should also be observable in a simple condition stripped of the complex contextual characteristics of the above situations. To this end, we conduct a controlled laboratory experiment applying the investment game [4] in which two players send money back and forth within or across borders. This setting mirrors a stylized decision situation like the ones in our introductory examples. An investor has to take a risky investment decision in a foreign country that involves transferring money to the investee who may or may not repay a due share of the amount received to the investor. The investor does not know the investee’s response and expectations. How to deal with this uncertainty? If standards within a country exist—i.e., investments and beliefs on investments are well calibrated—investors and investees might apply the within-country standard also to foreigners. If these standards differ between countries, however, problems similar to the introductory examples are bound to arise. Our study allows analyzing these phenomena and is the first controlled investigation on the calibration of behavior and beliefs in an inter-country setting.

To understand the (mis)alignment phenomenon we pursue the following research agenda: First, do we observe transfer standards—i.e. investments and beliefs on investments are well-calibrated—to exist within the pools of participating subjects from different countries? Second, do the standards differ substantially between these subject pools? Third, do we find negative discrimination in investment behavior or are foreigners treated alike regardless of their origin? Fourth, are beliefs miscalibrated between countries, i.e., are beliefs on foreigners’ investments and back-transfers influenced by home-country standards and therefore not adapted accordingly?

Our study uses the methodology of experimental economics to analyze un-calibrated beliefs in interactions with foreigners. It relates to the pioneering cross-cultural paper by [5] as well as to [6],[7], who further developed the research methodology. A large body of cross-country experimental studies exists by now; see the reviews by [8] and [9], see also [10],[11],[12] with several cross-country experimental investment game studies, c.f. [13]. Our paper substantially differs from these studies as we analyze actual inter-country interactions while in cross-cultural studies behavior within subject-pools (countries) is compared across countries. Only a small number of inter-country investment game studies exists. A controlled study on the calibration of behavior and beliefs in an inter-country setting is missing, however—as are surveys or field studies. Our study, thus, is the first one to investigate this important topic.

Our behavioral and belief data are based on 400 participants from three subject pools with different cultural backgrounds (Germany, Israel, Palestine) playing the investment game in two studies. Participants interact with experimental subjects within their own country (within-subject-pool interaction) as well as with those of foreign countries (inter-subject-pool interactions). For simplicity, we refer to Germany, Israel, and Palestine as countries having in mind that the Westbank is not part of an independent state. The research strategy of our study on how well behavior and beliefs in an inter-country setting are calibrated requires not only within- and across-border interactions of experimental subjects but also eliciting beliefs on fellow-country as well as on foreign-country participants’ choices. Within-border data are not sufficient because subjects would interact *within* their own country and behavior and beliefs can be compared *across* countries only. Choices and beliefs towards foreigners might, however, deviate from decisions and beliefs towards fellow-country members [13].

We chose to study our research questions in Germany, Israel and Palestine. The three countries are pertinent to our research goal. Not only are within-country standards likely to deviate between the countries due to differences on several dimensions (like e.g. religious background, country value scores, language, geographic location), participants can also be expected to have distinct perceptions of each other. Israelis and Germans share a difficult historical past, Israelis and Palestinians are engaged in a long-lasting and still ongoing conflict, and the media provides regular information on all three countries. We analyze the compatibility of beliefs and actions by comparing investors’ transfers and investees’ expectations within and across student subject pools from Bonn University, Germany, The Hebrew University of Jerusalem, Israel, Bethlehem University or Al-Quds University, Palestine.

For our study, it is important that we compare a very similar class of subjects from the three countries. Choosing university students makes it more likely that all subjects share similar levels of education and a middleclass background. However, cultural, economic, and other differences between the selected subject pools exist, which we cannot control for and which might lead to different transfer levels and expectations across subject pools. This, however, is intentional and not a caveat to our study that does not aim at explaining the different standards between the subject pools. It rather intends to explain un-calibrated beliefs on foreigners’ behavior by the investment standards prevalent within the home-country subject pool.

## Materials and Methods

In this Section we will first explain the experimental design followed by a detailed description of our experimental procedure. We then report on participants’ societal/cultural background and, finally, we illustrate our inter-country research methodology.

### Experimental design

In our investment game experiment, the investor was named ‘Person A’ and the investee ‘Person B’ in order to not induce additional framing effects. Each investor is paired once with a fellow-country investee and two times with a foreign one. In each matching, both investors and investees are endowed with an initial endowment of 10 Experimental Currency Units (ECU). The investor can transfer any part or nothing of his endowment to the investee while the investee pockets her endowment. The experimenter triples the transfer, and the investee can return any amount or nothing of the tripled investment to the investor. While an investor states the amount he transfers to the investee, the paired investee states for each of the tripled transfers the amount she returns to the investor. We also asked investors and investees to state their beliefs on how much each of their paired counterparts would transfer or return, respectively. Beliefs were not incentivized. The investee’s role is that of an allocator who distributes the tripled transfer between herself and the investor without having to expect any reaction of the latter. We, therefore, will call the investee ‘allocator’ in the remaining part of the paper.

Each German, Israeli, or Palestinian participant plays a one-shot investment game with one member of his/her own subject pool and with one member of each of the other two subject pools. No feedback was given before the end of the experiment. In Study 2 we analyze behavior in the first round only, thus relying on truly independent observations. Here, in each of the sessions one third of the participants first played a bilateral one-shot investment game with members of their own subject pool, the other two thirds of the participants were evenly matched with the two foreign subject pools.

### Experimental procedures

After showing up for the experiment, participants at each location randomly drew a personal identification code and a card indicating the number of their work place. The personal identification code corresponded to our coding system guaranteeing full anonymity by a double-blind procedure. The instructions were read aloud by a native experimenter giving detailed explanations on the experiment and the procedure. Questions were answered in private. We took great care to ensure that subjects understand the game and the underlying incentive structure by only proceeding in the experimental protocol after all subjects had correctly answered six control questions (see the English translation of instructions and control questions in S1 File, Section C. The materials in Hebrew, Arabic, and German are available from the authors upon request). Then, investors made their transfer choices and allocators wrote down their return decisions for all potential transfer values on decision sheets/screens displaying their counterparts' subject-pool affiliations: Bonn University (Germany), The Hebrew University of Jerusalem (Israel), Bethlehem University or Al-Quds University (Westbank, Palestine). Investors and allocators were also asked to state their beliefs on the same decision sheet/screen. For each of the three matchings, subjects were provided with a separate sheet/screen. Subjects received no feedback about their payoffs before the very end of the entire experiment.

Due to organizational constraints, we were not able to run the experiments simultaneously at all three locations. Therefore, each participant received the show-up fee at the end of a session. The outcome-related payoff was paid about a week later, i.e. after all sessions had been run and the chief experimenter from Bonn University had collected the data, computed the payoffs, and had transferred this information to the local experimenters. Subjects were informed at the end of a session about the exact date to pick up their final payments, which they finally did at their local experimenters. More details of the experimental procedure are provided in S1 File, Section A.

The experiments were run according to the ethics standards of the experimental economics profession that does not allow deception. Given these standards, and that our decision experiments do not involve any medical treatment or alike the approval by an ethics committee or institutional review board was not required, neither in the countries nor at the institutions where the experiments were run.

### Participants: subject pools and their societal/cultural background

In total, 400 students from various faculties participated in our experiment. Participants in Study 1 are 90 students, 30 each from Bonn University, The Hebrew University, and Bethlehem University. In Study 2, again students from Bonn University and Hebrew University took part. The Palestinian participants are students of Al-Quds University located in Abu-Dis close to Jerusalem in the Westbank. In total, 310 subjects took part in Study 2; 95–108 per country.

We controlled for participants' nationalities in order to exclude foreign players from national subject pools. In Jerusalem, Israeli Arab students did not participate in the experiment. We also tried to balance age and gender in each sample to guarantee similar conditions in each university. Each participant joined the experiments only once

Due to our research agenda, on the one hand it is important to investigate a similar class of subjects from the three countries. Choosing university students makes it more likely that all subjects share similar levels of education and a middleclass background. On the other hand, diversities between the selected subject pools exist like religious, economic, language, and societal differences, see Table 1 based on [14],[15],[16] and [17].

**Table 1:** Characteristics of the three locations.

	Germany	Israel	Palestine
**GDP/per capita (PPP)**	$39,500 (2013)	$36,200 (2013)	$2,900 (2008)
**Literacy**	99%	97.10%	95.30%
**Religious background**	Predominantly Christianity	Predominantly Judaism	Predominantly Islam
	Christian: 68% (equal shares of Protestants and Catholics)	Jewish: 75.1%	Muslim: 93% (predominantly Sunni)
	Muslim: 3.7%	Muslim: 17.4%	Christian: 6%
	Other: 28.3%	Christian: 2%	Other: 1%
		Druze: 1.6%	
		Other: 3.9%	
**Societies**	Individualistic, Low context	Individualistic, Low context	Collectivistic, High context
**Language**	German	Hebrew	Arabic
**Geographic location**	Western Europe	Middle East	Middle East

Note: Numbers taken from the CIA World Factbook (https://www.cia.gov/library/publications/the-world-factbook/, last access 05-28-2016).

### Research methodology: inter-country experiments

Inter-country and cross-country studies share many features like language, experimenter, and currency effects. In the following we discuss how we deal with these issues; for a detailed description, see [5–7]. An additional challenge in inter-country experiments like ours is that interactions between subject pools are interrelated and would require simultaneous sessions in all countries involved. Due to organizational constraints, we were not able to run the experiments simultaneously at all three locations. We also will explain our solution to this problem.

#### Language effects

All subjects received exactly the same instructions and materials with the only difference that they were provided in the respective native languages. Otherwise, behavioral disparities between countries may be caused by perceptual differences due to the material and are not attributable to country-specific characteristics. See e.g. [18] for rather large effects on decisions and beliefs in only minimally differently framed social dilemma experiments. Using the respective language, i.e., Arabic, German or Hebrew, ensured that randomly selected students at the different locations could participate without any further restrictions. Using only students fluent in, e.g., English, might have led to selection problems. Furthermore, recent studies show that subjects’ behavior is significantly influenced if instructions are not in the corresponding native language [19],[20]. To ensure an accurate translation of the instructions, we applied the back-translation procedure [21].

#### Experimenter effect

Different experimenters may conduct the experiment in slightly different ways. We therefore briefed all local experimenters in the same way to ensure the same application of the procedural protocol at all locations. In both studies, experimenters were personally instructed. In addition they received a detailed procedural script and an extensive instruction manual. In Study 1, experimenters at the different locations organized and run the sessions on their own. The organization of Study 2 was more complex given the higher number of observations and the larger number of participants that needed to be matched. Therefore, members of the Bonn team instructed the local experimenters at the respective locations and were available during the sessions.

#### Currency effects

Using local currencies during the experiments would have resulted in different ranges of numbers and different nominal payoffs, which might have biased the perceptions of endowments and transfers. To avoid this we used an artificial currency “ECU” (experimental currency unit) for describing and conducting the game. Only at the very beginning of the instructions we informed subjects that the ECU would be converted into real money with a fixed exchange rate at the end of the experiment. Thereafter, only the term ECU was used in the instructions. In addition, to ensure comparable stake sizes we calibrated total payments and show-up fees based on hourly wages of experimental subjects and/or student helpers in the respective subject pools.

#### Inter-country challenges

In both studies, it was not possible to run simultaneous internet-based real-time sessions due to various organizational constraints. To overcome these challenges we combined several experimental features known from the literature or developed by ourselves [22]. First, we had to solve the interdependence issue of sequential choices as an allocator in one subject pool cannot return money to a foreign investor in the second stage of the game unless she knows how much the investor has transferred to her in the first stage. By applying the strategy method [23] we organizationally disconnected the second from the first stage of the game. Having the allocator state her back-transfer for each possible transfer of the investor, the sequential two-person two-stage game is converted into a two-person normal-form one-stage game for each player. We now were able to run the experiment at slightly different points in time at the three locations independently of each other with the time lag being three to five days.

Second, we used a remote-control organization by designing an experimental procedure that guaranteed equivalent experimental conditions in all three subject pools. We developed a coding system by matching players across subject pools ex ante for each of the decisions, thus predefining the order of matchings. Organizing all experimental procedures and technical issues as well as the time coordination was done at one location.

Third, the Bonn experimenter team provided the coded decision sheets and all other materials in the three native languages for the pen-and-paper Study 1 where local experimenters were in charge of organizing and running the sessions. In Study 2, where local experimenters run the sessions as well but Bonn experimenters were available, we used the experimental software z-Tree [24] to facilitate the matching procedure, data collection and data analysis. Bonn provided the z-Tree program in all three languages.

## Results

To pursue our research agenda we are interested in two main variables: first, the monetary transfer investors make towards a foreign or fellow-country allocator who may or may not return any money; second, the belief of the allocator on the investor’s transfer.

### Descriptives

In Study 1, in each location we gathered data from 15 investors and 15 allocators (average age: Germany 22.5, Israel 25.4, Palestine n.d.; percentage of females: Germany 43, Israel 47, Palestine 40). Each participant made three consecutive decisions.

In Study 2, we collected data for up to 54 investors and 54 allocators in each location (average age: Germany 22.8, Israel 24.1, Palestine 20.5; percentage of females: Germany 56, Israel 44, Palestine 33). In each of the matchings in the second study, we have up to 18 independent observations. See Tables A and B in S1 File.

Table 2 gives an overview about the average transfer investors made within their own subject pool and towards participants of foreign subject pools. The table also shows the average beliefs allocators had on investors’ transfers within and across borders. Averaged over all subject pools, transfers are rather high (50 percent of investors’ endowments). Allocators’ average belief on investors’ transfers is somewhat higher (53 percent of investors’ endowments).

**Table 2:** Investors’ transfers and allocators’ beliefs.

		Allocator subject pool		
	Investor subject pool	Israelis	Palestinians	Germans
**Study 1**				
**Transfers, ECU (SD)**	Israelis (*I*)	3.9 (3.4)	2.9 (3.4)	3.9 (3.1)
	Palestinians (P)	6.2 (2.5)	6.7 (2.3)	6.9 (1.6)
	Germans (*G*)	5.2 (3.5)	5.6 (3.3)	4.4 (3.3)
**Beliefs, ECU (SD)**	Israelis (*I*)	4.2 (3.3)	6.9 (2.8)	4.5 (3.5)
	Palestinians (*P*)	3.7 (3.6)	7.7 (2.8)	4.7 (3.3)
	Germans (*G*)	4.8 (3.4)	6.5 (2.6)	4.8 (3.1)
**Study 2**				
**Transfers, ECU (SD)**	Israelis (*I*)	4.3 (3.3)	4.1 (3.2)	6.3 (2.5)
	Palestinians (*P*)	6.5 (3.1)	8.0 (2.3)	7.6 (2.0)
	Germans (*G*)	7.1 (2.5)	5.3 (3.4)	6.0 (2.6)
**Beliefs, ECU (SD)**	Israelis (*I*)	4.3 (2.1)	5.2 (3.1)	4.1 (2.3)
	Palestinians (*P*)	3.8 (1.8)	6.8 (2.5)	4.9 (3.1)
	Germans (*G*)	4.8 (2.6)	4.8 (1.6)	4.1 (2.4)

### Transfer standards within countries

**Fig 1:**
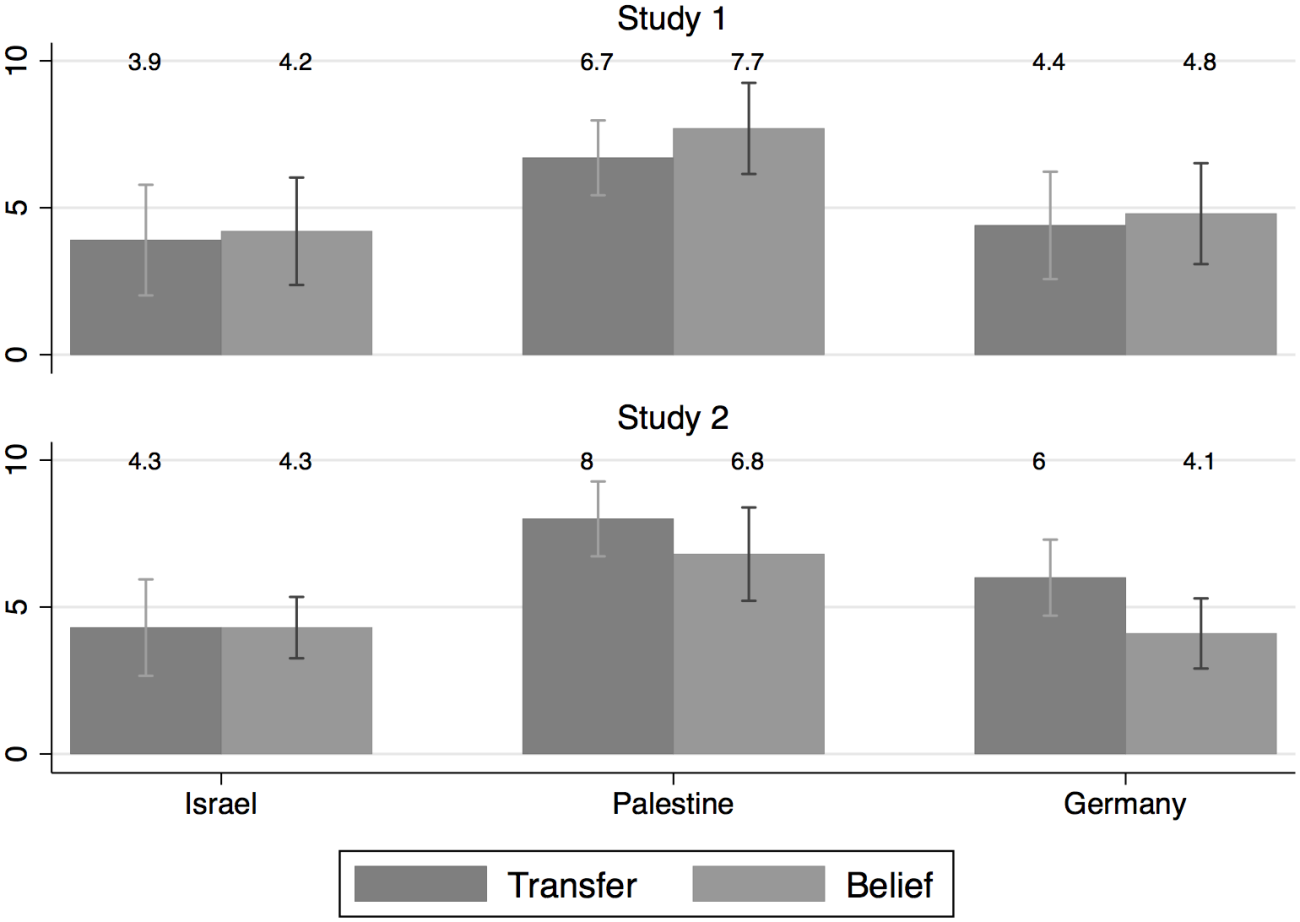
Mean transfers, mean beliefs about transfers, and standard errors in both studies. All values are in ECU and are given for transfers and beliefs within the fellow-country subject pools. See also Table 2.

Following our research agenda, we first investigate whether transfer standards exist within countries, i.e. whether allocators correctly anticipate investors’ behavior within their own subject pool. We find allocators’ beliefs to match fellow-country investors' behavior very well (Fig 1). For mean beliefs about transfers in both studies in all combinations of allocator and investor subject pools see Figure A in S1 File.

In Study 1, transfers and beliefs do not differ significantly within the three subject pools (all comparisons with *p ≥* .*323*, two-sided Fisher-Pitman permutation tests for two independent samples—FP in the following). For our Israeli and Palestinian samples the finding that allocators correctly anticipate fellow-country investors’ transfer behavior is robust across studies (Study 2: *p ≥* .*218*, two-sided FP). In Germany allocators significantly underestimate fellow-investors’ transfers in Study 2 (*p <* .*05*, two-sided FP). Overall, we find significant correlations between mean transfers and individual beliefs (both studies with *r>0*.*34* and *p<0*.*05*, Spearman correlation; see Table C in S1 File for all *p*-values of the pairwise comparisons of all relevant variables investigated in this study).

*Summary*: *In general*, *we find transfer standards to exist within countries as transfers and beliefs do not differ significantly within the three subject pools*.

### Different transfer standards across countries

Our second research question is concerned with whether average transfer levels differ between the three subject pools, and if so whether these disparities are also reflected in allocators’ beliefs on investors’ transfers. We find significant differences in transfers between the different subject pools in Study 1 (*p <* .*05*, two-sided Kruskal-Wallis-test—KWT in the following) as well as in Study 2 (*p <* .*01*, two-sided KWT). Pair-wise comparisons show that transfers within the locations are significantly higher in the Palestinian subject pool than in the German and Israeli ones (*p <* .*05*, two-sided FP for all comparisons in both studies). Furthermore, we observe a weakly significant difference between transfers within Israel and Germany in Study 2 (*p =* .*099*, two-sided FP). These different transfer levels are also reflected in beliefs, as they differ significantly between the locations (both studies: *p<0*.*01*, two-sided KWT).

Summary: Transfer standards are different across countries in that transfer levels differ between the three subject pools and these disparities are reflected in the allocators’ beliefs on investors’ transfers.

### Discrimination between countries

**Fig 2:**
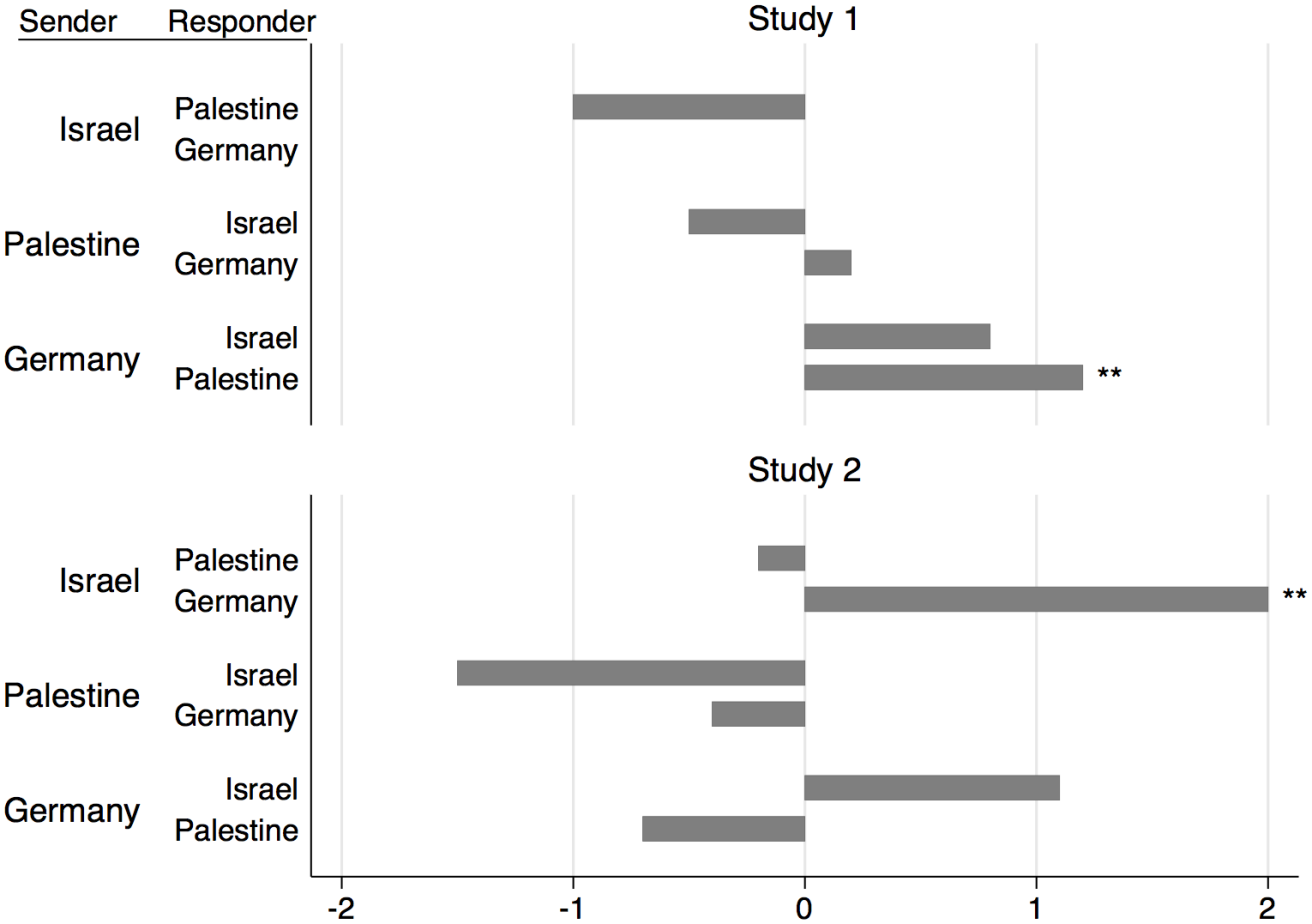
Difference between mean transfer to a foreign subject pool and mean transfer to the fellow-country subject pool. For each matching, the first column indicates the investor’s, the second the allocator’s location. Significant differences between transfers to a foreign and the fellow-country subject pool are denoted by stars (* *p≤*.*10* ** *p≤*.*05*; based in Study 1 on two-sided Fisher-Pitman permutation tests for paired replicates and in Study 2 on two-sided Fisher-Pitman permutation tests for two independent samples).

Investigating our third research question on discriminative behavior we obtain a further robust result: No systematic negative discrimination of foreigners is observed. In all countries, we do not find significantly lower transfers to foreigners compared to own subject-pool transfers (Fig 2).

We observe, however, significantly higher transfers to Palestinian subjects from Germans in Study 1 and significantly higher transfers to Germans from Israel in Study 2 (Study 1: *p <* .*05* two-sided Fisher-Pitman permutation tests for paired replicates—FPPR in the following; Study 2: *p <* .*05*, two-sided FP).

We also do not find evidence for systematic negative discrimination in investors’ beliefs on allocators’ backtransfers. Pairwise comparisons reveal only for Israeli senders significant differences in beliefs on backtransfers from own subject-pool subjects and subjects from Palestine in Study 1 (p = .046 FPPR) and subjects from Germany in Study 2 (p = .073 FP). In both cases senders expect higher backtransfers from the foreign subject-pool. Taking the average expected backtransfers from foreign subject-pools we find for all locations no significant differences to expected backtransfers from the corresponding home subject-pool (Study 1: all p>.16 FPPR; Study 2: all p>.21 FP); see Figure B and Table C in S1 File.

The non-existence of systematic negative differentiation is in line with inter-country investment game studies that use an experimental design similar to [4] as we do where transfers and backtransfers are not restricted. See the discussion section for a detailed comparison of our findings with the literature.

*Summary*: *We do not find evidence for systematic negative discrimination of foreigners*.

### (Mis)calibration of beliefs

**Fig 3:**
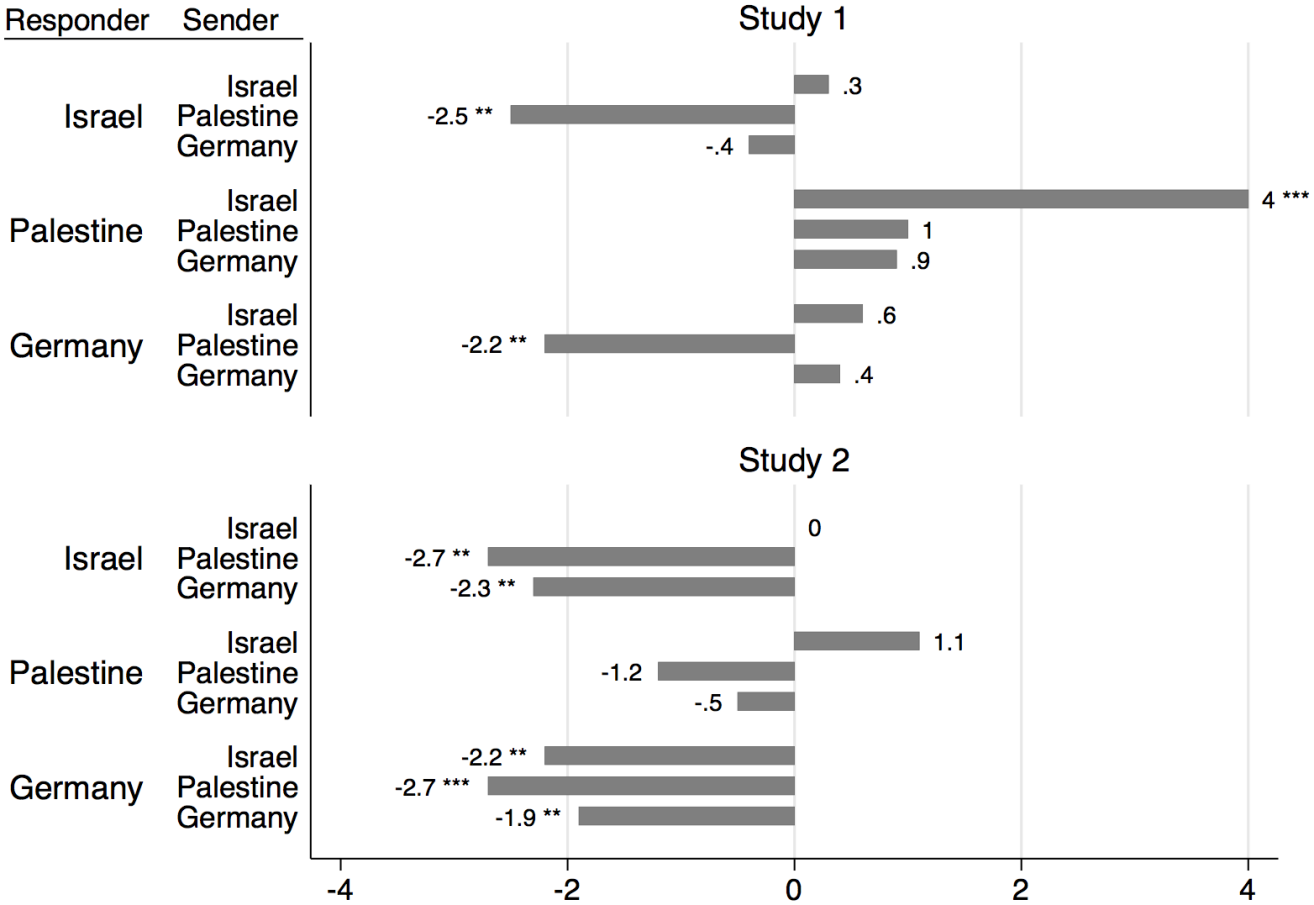
Difference between expected and actual transfer. The first row of locations indicates the allocator’s location and the second column the one of the investor. Significant differences between expected and actual transfers are denoted by stars (* *p≤*.*1* ** *p≤*.*05* *** *p≤*.*01*; two-sided Fisher-Pitman permutation tests for two independent samples).

Even though we do not observe negative discrimination of foreigners, it is unclear which transfer levels allocators expect and if those expectations were accurate. In fact, we observe that foreign allocators’ expectations are rather un-calibrated in both studies (Fig 3). Fig 3 provides all differences between expected and actual transfers, as well as the level of significance if they differ significantly.

German and Israeli allocators significantly underestimate the transfers from Palestinian investors (all comparisons of beliefs with actual transfers from Palestinians *p <* .*05*, two-sided FP). Moreover, in Study 2, Germans (Israelis) significantly underestimate transfers from Israelis (Germans). In Study 1, Palestinian allocators drastically overestimate transfers from the Israeli subject pool (*p <* .01, two-sided FP). In Study 2, beliefs are still higher, but the difference is no longer significant (*p =* .*395*, two-sided FP). In addition, as beliefs are biased by home-country transfer standards, subjects do not adapt them when facing a subject from a foreign subject pool: all pair-wise comparisons between beliefs on fellow-country participants’ and foreigners’ transfers do not differ significantly (Study 1: *p>*.*24*, two-sided FPPR; Study 2: *p >* .*22*, two-sided FP).

Finally, testing the absolute difference between beliefs and transfers reveals that expectations on foreign transfers are significantly less accurate than beliefs on same subject-pool investments (Study 1: *p <* .*05*, two-sided FPPR; Study 2: *p <* .*05*, two-sided FP).

We also analyzed whether investors’ beliefs on backtransfers are miscalibrated. And that is what we observe. In Study 1, expected backtransfers from Palestine to Israel (*p <* .*05*) and Germany (*p <* .*1)* are significantly lower than actual backtransfers (both two-sided FP). All other comparisons are either not significant (Palestinian investors in Study 2) or backtransfers are significantly overestimated (all other investors from own and foreign subject pools); see Figure B in S1 File. Contrary to what we found for allocators, the investor’s inaccuracy is not restricted to foreign backtransfers, but occurs within the own subject-pool as well. Testing the absolute difference between beliefs and average backtransfers shows that expectations on foreign backtransfers are not significantly less accurate than beliefs on own subject-pool backtransfers (Study 1: *p =* .*91*, two-sided FPPR; Study 2: *p =* .*66*, two-sided FP).

*Summary*: *Allocators’ beliefs are biased towards home-country transfer standards*. *Therefore*, *foreign allocators’ expectations are rather un-calibrated in both studies*. *Investor’s beliefs are miscalibrated as well*, *but this holds for own-country and foreign subject pools alike*.

## Discussion

Even though investment games enjoy widespread use in experimental economics (see the surveys by [25],[26], see also [27]) including a few papers on inter-country behavior (in addition to [28–32],[22] see the survey by [33], see also [34–37]) our study is the first controlled investigation on the calibration of behavior and beliefs in an inter-country setting. None of the previous studies elicited belief data except for [22],[30] who provide no analyses of the belief data, however. Moreover, our inter-country research seems to be pioneering work in the Westbank; we know of no other experiment that involves Palestinian participants interacting with foreign counter players except for [38] who, however, use virtual instead of real counter players and thus do not involve actual inter-country interactions.

The small number of experimental inter-country investigations may be due to the large organizational and administrative challenges associated with this kind of research. To reduce the difficulties some studies use participants of different nationalities at one place [39],[40], or do not translate the instructions to the native languages [41].

Our results partly differ from the previous literature. Therefore, it seems necessary to put our findings into perspective. In line with findings from two meta-analyses [42],[43] and own literature checks a differentiated view on the research area seems necessary to better understand the prevailing phenomena. We will proceed along our research agenda.

### Transfers within countries

We first focus on transfers within countries. Within our German subject-pool we observe a considerable amount of transfers (52 percent, Table 2), which is in line with the reported behavior of German investors in a survey on investment game experiments [26]; although transfers in our second study are on higher level. In Israel, we observed lower transfers (42 percent) than in [26], see also [44],[45],[46]. The latter three studies differ from ours in several respects. In [46] computerized allocators are used. In [44] and [45], allocators do not receive an initial endowment and the experiment involves transfers to specific ethnic/religious groups in Israel. Both factors might have induced more generous transfers. As to Palestine, we are not aware of any other investment game experiment we could compare our results with. Some investment game experiments in the Arabic States Kuwait, Oman, and the United Arab Emirates [47],[48] found transfers to be much lower than in Western countries, which is in contrast to our results from the Palestinian subject pools. Yet, the designs of [47] and [48], as well as the subject pools are hardly comparable to ours.

### Different transfer standards between countries

We found transfers and beliefs on transfers well calibrated in all three subject-pools. Our results for allocators seem to reflect the fact that participants are familiar with peoples’ behavior in their home country [49],[50]. This is in line with the hypothesis suggested by [5] that subject pools share a common idea about what constitutes a reasonable transfer and thus the subject-pool differences observed in [5] are related to different expectations across subject pools (countries) on what that choice actually is. However, due to missing belief data this conjecture was never tested. Other evidence is not as clear-cut; [51], for instance, observed that allocators’ beliefs match transfer behavior in the U.S. but not in China.

One might wonder why, compared to allocators, investors in our experiment are rather bad in correctly predicting their fellow-country counterparts’ choices. Our conjecture is that it has to do with the structure of the investment game: investors take a decision that entails an element of strategic risk. They might therefore need different strategies to rationalize their decisions, which may be reflected in their stated beliefs. Furthermore, while allocators’ beliefs are independent of their own decision (i.e. the size of their backtransfer), investors’ beliefs on backtransfers, in contrast, are not. For instance, for a transfer of 10 the range of beliefs on backtransfers differs compared to a transfer of 0. Given the heterogeneity in transfers within the subject pools and the possible variance in investors’ beliefs our sample size seems not large enough to tease out meaningful differences between the subject pools.

### No negative discrimination

A further robust result is that we do not find negative discrimination in any of the subject pools in our two studies neither in transfers nor in belief data of investors and allocators. This non-discrimination finding seems in contrast to the large body of literature on in-group/out-group bias [52] and in particular to the investment game studies in Israel [44],[45], and [53]. Yet, discrimination in experiments does not seem as prevalent as the previous literature has suggested. Two meta-analyses of experimental studies on different games [42,43] that overlap in only one third of the studies included show the effect size of discrimination to be rather small (0.252 in [42], 0.32 in 43]). Discrimination appears to vary depending upon the type of group identity being studied: [42] finds that it is weaker when subject pools are defined by the natural characteristic ‘nationality’ like in our study compared to when identity is artificially induced in the laboratory. Moreover, discrimination does not significantly differ between students and non-students [42].

To put our non-discrimination results into perspective, we checked those inter-country investment game studies that apply an experimental design similar to ours. We find that our results are supported: no evidence for discrimination towards foreign subject pools is reported in [28]-[32] and in two of the three subject pools in [22]. Consistent negative discrimination in all investigated subject pools is only found by [30] while in [22] evidence is reported for one of the three investigated subject pools only.

### (Mis)calibration of beliefs

(Mis)calibration of choices and beliefs is a new finding as belief elicitation in inter-country investment game experiments is practically non-existent. We also know of no studies on the compatibility of beliefs and actions in these kinds of experiments within Israel and Palestine. For Germany, there is additional evidence that subjects have well-calibrated beliefs about own-country transfer standards [54].

A plausible explanation for the miscalibration of beliefs is given by rule-rationality. People “adopt rules, or modes of behavior, that maximize some measure of total or average or expected utility, taken over all decision situations to which that rule applies” [55], see also [56]. Not adapting beliefs is an effective rule for most within-country every-day decision situations as the great majority of interactions is going on between fellow-country people but much fewer with foreigners. A finding by [57] supports this explanation: not readily having the necessary specific information at hand induces experimental participants to base their beliefs on their own behavior in a similar situation. Such an effect could be at work in our experiment in that subjects tend to take the transfer standards of their home country as a reference point.

A further reason for the observed miscalibration of the participants’ beliefs may be caused by their flawed perception of how own behavior in a trust environment is perceived by others, be it own-country people or foreigners; i.e. a disconnect exists of how the participant thinks he or she (his or her country) is perceived vs. how it is actually perceived. Such an analysis would imply to elicit second order beliefs, which we did not but which seems an important area for future research. Referring to our setting we believe, however, that the reference point argument above provides a good explanation for the allocators’ observed behavior. Our discussion of investor behavior calls for larger samples to test the above conjecture.

## Conclusion

We run two studies on bilateral intra- and inter-country investment game experiments played between Germans, Israelis, and Palestinians. Focusing on allocators’ behavior and eliciting their beliefs on fellow-country and foreigners’ transfers we found a notable constellation of calibrated and un-calibrated beliefs, which is robust over time. Within each country, transfer standards exist, which allocators correctly anticipate. However, across countries these standards differ. By attributing the standard of their own environment to the other countries, allocators are remarkably bad in predicting foreigners’ behavior.

We did not aim at explaining the different standards between the subject pools. We rather intended to explain un-calibrated beliefs on foreigners’ behavior by the standards prevalent within the home-country subject pool.

Humans’ ability to predict the behavior of people from their origin is probably linked to the learning process that we experience through a variety of daily interactions taking place within our own social environment [49],[50],[58]. Not adjusting to a specific situation might be an effective rule for most every-day decisions; when interacting with foreigners in inter-country settings, however, it leads to a miscalibration of beliefs. When one party encounters unexpected behavior by another party he/she might tend to believe that such behavior (whether positive or negative) is specific to the interaction ignoring the fact that such behavior might simply be due to dissimilarities among foreigners. The tendency to ignore this potential difference can be a source of misinterpreting motives in inter-country interactions that may induce mistrust, enhance conflict or impede its resolution as in our opening examples.

While it is reasonable to assume that similar effects like in our experiment will be found in comparable strategic interactions it ultimately remains an empirical question to demonstrate so.

## Supporting Information

S1 Data (ZIP)

S1 File (PDF)
