# Supplementary material for: In Wrong Anticipation - Miscalibrated Beliefs between Germans, Israelis, and Palestinians
Source: PLoS One. 2016 Jun 16;11(6):e0156998. doi: 10.1371/journal.pone.0156998 (PMC4911115; doi:10.1371/journal.pone.0156998)
Supplement: S1 File — (PDF) [file pone.0156998.s002.pdf]

SI: Supporting Information

"In wrong anticipation - Miscalibrated beliefs on trust between Germans, Israelis, and Palestinians"

by

Sebastian J. Goerg[1], Heike Hennig-Schmidt[2], Gari Walkowitz[3], Eyal Winter[4]

Section A Experimental procedure and participant payment

Section B Descriptives

Section C Instructions and control questions

Section D References

[1] Department of Economics, Florida State University, Tallahassee, USA, & Max Planck Institute for Research on Collective Goods, Bonn, Germany (sgoerg@fsu.edu).

[2] Laboratory for Experimental Economics, University of Bonn, Bonn, Germany & Department of Health Management and Health Economics, University of Oslo, Norway (hschmidt@uni-bonn.de).

[3] Corporate Development and Business Ethics, University of Cologne, Cologne, Germany (walkowitz@wiso.uni-koeln.de).

[4] The Center for the Study of Rationality, The Hebrew University of Jerusalem, Jerusalem, Israel (eyal.winter@mail.huji.ac.il).

Section A Experimental procedure and participant payment

All participants at all locations were instructed identically in their home language, instructors reading a script describing the task. The identities of matched participants were never revealed and all decisions were taken anonymously. Only the name of the counter player's university and his or her country of origin were provided and made salient.

Subjects were recruited by campus advertisements, e-mails and the online-recruiting system ORSEE [S1] announcing a monetary reward for participation in a decision-making task. Each session either comprised trustors or trustees and lasted for about 90 minutes including reading the instructions.

We run the experiments with Israelis in Ratio Lab, The Hebrew University of Jerusalem's experimental laboratory at the Center for the Study of Rationality. In Germany, sessions were run in bonneconlab, the University of Bonn's Laboratory for Experimental Economics. In both laboratories, participants at their workstations were separated by partitions. In Palestine, we run the experiments in large classrooms. In both Palestinian locations, participants were sitting far apart from each other such that anonymity of decisions was guaranteed. Study 1 was a pen-and-paper experiment. In Study 2, we used the software z-Tree [S2]. Yet, due to technical problems, we had to apply a paper-and-pencil procedure in Palestine in Study 2 as well. We printed the screenshots of all computer screens and handed them over to the experimental subjects for entering their decisions, beliefs and answers to the additional questions. All texts that appeared on the computer screens, instructions, as well as decision sheets and all other material were translated into the respective language.

Student participants who joined our economic decision experiments in Bonn and Jerusalem were recruited from a large subject pool consisting of subjects who signed up for taking part in economic decision laboratory experiments conducted at the respective institutions. When signing up the first time for an experiment (which might not be identical

with the present one), subjects were informed about the guidelines in experimental economics studies and about the specific rules at the respective institution. Having been informed about the rules, subjects provided written consent that they agree to take part in experiments conducted at the respective institution. The consent forms for general participation are stored in the respective laboratories. In Al Quds and Bethlehem University, subjects were ad-hoc recruited by the local staff of the facilities via public announcements. Responsible for the recruiting process were our local project leaders. After recruitment, subjects registered for the experiment. During the registration, subjects were informed about the rules and provided consent by eventually signing up for the experiment. All local project leaders and supporters approved our procedure, which strictly followed the ethics code in our field. As mentioned above, a specific approval for our study was not required.

The exchange rate was 1 ECU = 0.50 USD in each subject pool in Study 1 and 1 ECU = 0.25 Euro (1.25 NIS) in Study 2. A player's total earning was the payoff sum over all three matchings plus a fixed show-up fee [5 USD for Israelis and Germans and 3 USD for Palestinians in Study 1 and 5 Euro (25 NIS) in Study 2]. Subjects were paid in their national currency – NIS in Israel and Palestine, and EURO in Germany.

On average, subjects in Study 1 earned 23.9 USD (Israelis 24.6 USD, Palestinians 24.0 USD, and Germans 23.2 USD). In Study 2, subjects on average earned 17.6 USD (Israelis: 14.6 USD, Palestinians: 19.5 USD, and Germans 18.9 USD) plus the respective show-up fees.

Section B Descriptives

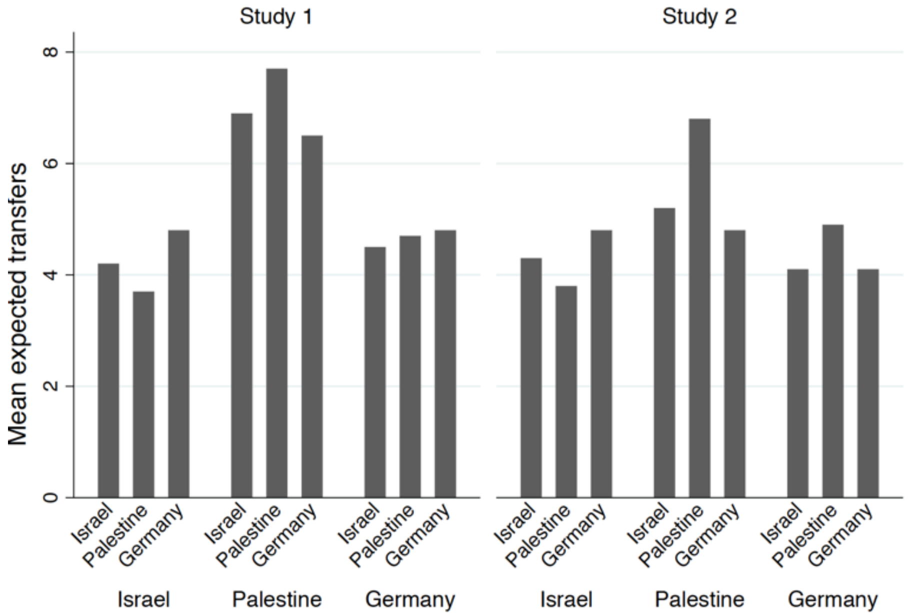

Figure A Mean beliefs about transfers in both studies. The values are given for all combinations of responder and sender countries.

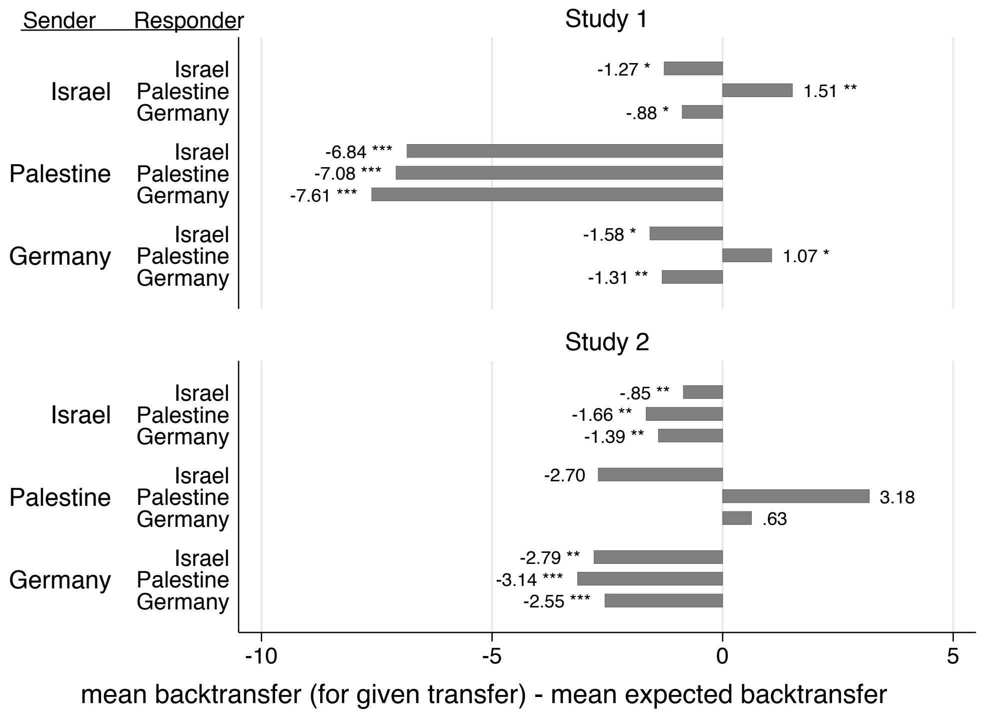

Figure B Difference between mean back transfer (for a given transfer level) and mean expected back transfer. The first row of locations indicates the investor's location and the second column the one of the allocator. Significant differences between expected and average back transfers are denoted by stars (* *p≤.1* ** *p≤.05* *** *p≤.01*; based in both studies on two-sided Fisher-Pitman permutation tests for two independent samples).

Table A Number of subjects in each matching in Study 1

Location	# Subjects					
	Sender's transfers to (in the following order)			Responder's belief (in the following order)		
	Germany, Israel, Palestine	Israel, Palestine, Germany	Palestine, Germany, Israel	Germany, Israel, Palestine	Israel, Palestine, Germany	Palestine, Germany, Israel
Israel	5	5	5	5	5	5
Palestine	5	5	5	5	5	5
Germany	5	5	5	5	5	5

Note: Every subject made a decision in three different matchings. No feedback was provided between decisions. Thus, in each location we gathered data from 15 senders and 15 responders. The observations are treated as dependent.

Table B: Number of subjects in each matching in Study 2

Location		# Subjects	
Sender	Responder	Sender's transfer	Responder's belief
Israel	Israel	18	18
Israel	Palestine	18	18
Israel	Germany	18	18
Palestine	Israel	15	11
Palestine	Palestine	15	12
Palestine	Germany	18	18
Germany	Israel	18	18
Germany	Palestine	18	17
Germany	Germany	18	18

Note: In each location we gathered data for up to 54 senders and 54 responders. In each matching, we have up to 18 independent observations. As we had to rely in our Palestinian subject pools on pen and paper some responses were incomplete or unreadable. We excluded these subjects from our analyses. If a participant was matched with a subject whose responses were missing we used the decision of another randomly chosen subject in the same role and the same matching to determine the respective participant's payoff.

Table C: *p*-values for pairwise comparisons of all relevant variables

a. Comparison of transfers and beliefs within subject pools: The table gives p-values of two-sided Fisher-Pitman permutation tests between transfers and beliefs within one subject pool

	Study 1			Study 2		
	Germany	Israel	Palestine	Germany	Israel	Palestine
Israel		.870			1	
Palestine			.323			.218
Germany	.776			.039		

b.1 Comparison of trust standards between subject pools (transfers): The table gives p-values of two-sided Fisher-Pitman permutation tests for the comparison of within-subject-pool transfers between two subject pools

	Study 1			Study 2		
	Germany	Israel	Palestine	Germany	Israel	Palestine
Israel	.744		.019	.099		.001
Palestine	.042	.019		.031	.001	
Germany		.744	.042		.099	.031

b.2 Comparison of trust standards between subject pools (beliefs): The table gives p-values of two-sided Fisher-Pitman permutation tests for the comparison of within-subject-pool beliefs between two subject-pool

	Study 1			Study 2		
	Germany	Israel	Palestine	Germany	Israel	Palestine
Israel	.651		.006	.836		.011
Palestine	.015	.006		.012	.011	
Germany		.651	.015		.836	.012

c. Investigating discrimination: The table gives p-values of two-sided Fisher-Pitman permutation tests for the comparison between transfers to own subject pool and foreign subject-pool subjects. In study 1, the Fisher-Pitman permutation test for paired replicates is applied. All significant p-values are for incidences of positive discrimination, i.e., foreign subject-pool members receiving higher transfers.

	Study 1			Study 2		
Sender	Germany	Israel	Palestine	Germany	Israel	Palestine
Israel	1		.236	.047		.879
Palestine	.808	.519		.614	.150	
Germany		.475	.045		.252	.512

d. Investigating discriminating beliefs: The table gives p-values of two-sided Fisher-Pitman permutation tests for the comparison between expected backtransfers from own subject pool and foreign subject-pool subjects. In study 1, the Fisher-Pitman permutation test for paired replicates is applied. All significant p-values are for incidences of positive discrimination, i.e., foreign subject-pool members receiving higher transfers.

	Study 1			Study 2		
Sender	Germany	Israel	Palestine	Germany	Israel	Palestine
Israel	.672		.046	.073		.884
Palestine	.968	.223		.702	.822	
Germany		.569	.155		.267	.857

Section C Instructions and control questions

Introduction

Thank you for participating in our international experiment. Please read the following instructions carefully. If you have any questions, please raise your hand. We, then, will come to you and answer your question personally. During the whole experiment it is very important that you do not communicate with other participants of the experiment.

In this experiment you can earn money. Your individual earnings depend on your own decisions and on the decisions of other participants in the experiment. Your payments will first be stated in points with 1 point = 0,25 Euro [1.25 NIS]. After the experiment has finished your payment will be converted into Euro [NIS] and paid to you in cash. In addition to your earnings from the experiment, you will receive a fixed amount of 5 Euro [25 NIS].

At the beginning of the experiment, you have drawn a personal code number. On presenting this code number you will later get your payments from this experiment. Please be careful keeping the code number! Without code number we are not able to provide your earnings.

- You will receive the fixed amount of 5 Euro [25 NIS] today right after the experiment.
- You will receive your individual payment at the date stated on the sheet of paper that we have distributed already.

During the experiment you are interacting with other participants assigned to you. Neither you nor anybody else will ever be told who these other participants are.

After this introduction we will ask you to answer some test questions to check whether you understood the experimental procedures. Then the experiment itself will start. After the experiment, we will ask you to answer some further questions

General design of the experiment

The experiment consists of several parts. In the following, we explain the design of part one. During the experiment two participants are interacting with each other. These participants are called Person A and Person B from now on. All persons that are participating in the experiment just now belong to the same type of persons, i.e. they all are either Person A or Person B. All participants in this experiment – whether they are Person A or Person B – receive the same experimental instructions.

At the beginning, each Person A and each Person B receives an initial endowment of 10 points. Person B puts this money aside. She will be paid this amount later in addition to her experimental earnings.

Decision procedure for Person A

Person A can now choose to transfer any integer part of his initial endowment from 0 to 10 points to Person B. Each amount transferred by Person A is tripled by the experimenter, i.e., Person B receives three times the amount chosen to be transferred by Person A.

An example: If Person A transfers 4 points, Person B receives 12 points. If Person A transfers 6 points, Person B receives 18 points.

Decision procedure for Person B

Person B decides how many points she wants to return to Person A. Person B is not informed about the amount Person A actually transferred. Therefore, Person B has to decide for each amount that Person A can possibly transfer to her how much she will return to Person A. Person B can return any integer from 0 to 30 at most. The amount Person B returns is not multiplied.

An example: If Person A transfers 4 points, Person B receives 12 points. Person B can then return 12 points at most. If Person B returns 4 points, Person A receives 4 points. If Person B returns 6 points, Person A receives 6 points.

The decision table for Person B looks as follows:

If Person A transfers to you (in points):	You receive:	Which amount do you want to return to Person A?
0	0	
1	3	
2	6	
3	9	
4	12	
5	15	
6	18	
7	21	
8	24	
9	27	
10	30	

Now the first part of the experiment for both persons has finished.

Calculating individual payments

For calculating individual payments the decisions of the paired Persons A and B are compared and Person A's actual transfer is assessed. The decision Person B has taken for exactly this amount is relevant for payment.

Individual payments for Persons A and B are calculated in the following way:

Individual payment Person A:

Initial endowment = 10 points
– amount transferred to Person B (in points)
+ amount returned from Person B (in points)
**= individual payment Person A**

Individual payment Person B:

Initial endowment = 10 points

+ tripled amount, transferred by Person A
– amount returned to Person A
**= individual payment Person B**

Example: Person A transfers 4 points to Person B. Person B then receives 12 points. Person B decides to return 5 points.

Person A's individual payment:

Initial endowment	10 points
- amount transferred	- 4 points
+ amount returned from Person B	+ 5 points
**= individual payment Person A**	**= 11 points**

Person B's individual payment:

Initial endowment	10 points
+ tripled amount, transferred by Person A	+ 12 points
– amount returned to Person A	- 5 points
**= individual payment Person B**	**= 17 points**

Additional information on the experimental procedure

- The experiments will not start unless all participants have answered all test questions correctly.
- Each participant is informed on the computer screen whether he/she is assigned the role of Person A or Person B.
- Each participant is informed on the computer screen about the name of the university and the country it's located in, where the paired Person A or B is studying and is taking his/her decisions.

Do you have any questions?

Test questions:

- If Person A transfers 10 points, how many points does Person B receive from Person A?
- What is the maximal amount in points, Person B can return if Person A transferred 0 points?
- If Person A transfers 0 points, how many points does Person B receive from Person A?
- What is the maximal amount in points, Person B can return if Person A transferred 10 points?

- What is Person A's individual payment in points if he transferred 5 points and Person B returned 7 points?
- What is Person B's individual payment in points if Person A transferred 3 points and Person B returned 5 points?

Instructions Study 1

Instructions in the pen-and paper Study 1 were slightly different. Participants received a booklet containing tables similar to the screens for test questions and decisions for Persons A and B. Also sheets containing the text of the intermediate screens for all three parts of the experiment were included in the booklet.

Screenshots

Decision screen: Person A (Sender)

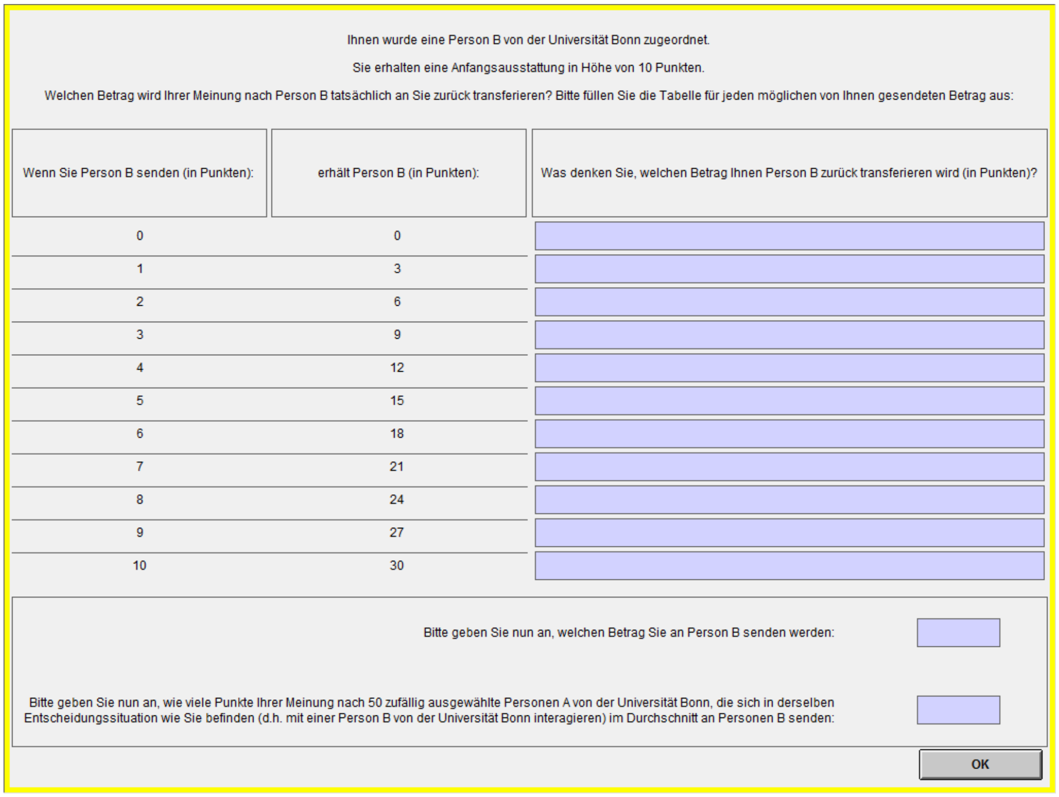

Ihnen wurde eine Person B von der Universität Bonn zugeordnet.
Sie erhalten eine Anfangsausstattung in Höhe von 10 Punkten.
Welchen Betrag wird Ihrer Meinung nach Person B tatsächlich an Sie zurück transferieren? Bitte füllen Sie die Tabelle für jeden möglichen von Ihnen gesendeten Betrag aus:

Wenn Sie Person B senden (in Punkten):	erhält Person B (in Punkten):	Was denken Sie, welchen Betrag Ihnen Person B zurück transferieren wird (in Punkten)?
0	0	
1	3	
2	6	
3	9	
4	12	
5	15	
6	18	
7	21	
8	24	
9	27	
10	30	

Bitte geben Sie nun an, welchen Betrag Sie an Person B senden werden:

Bitte geben Sie nun an, wie viele Punkte Ihrer Meinung nach 50 zufällig ausgewählte Personen A von der Universität Bonn, die sich in derselben Entscheidungssituation wie Sie befinden (d.h. mit einer Person B von der Universität Bonn interagieren) im Durchschnitt an Personen B senden:

OK

Decision screen: Person B (Responder)

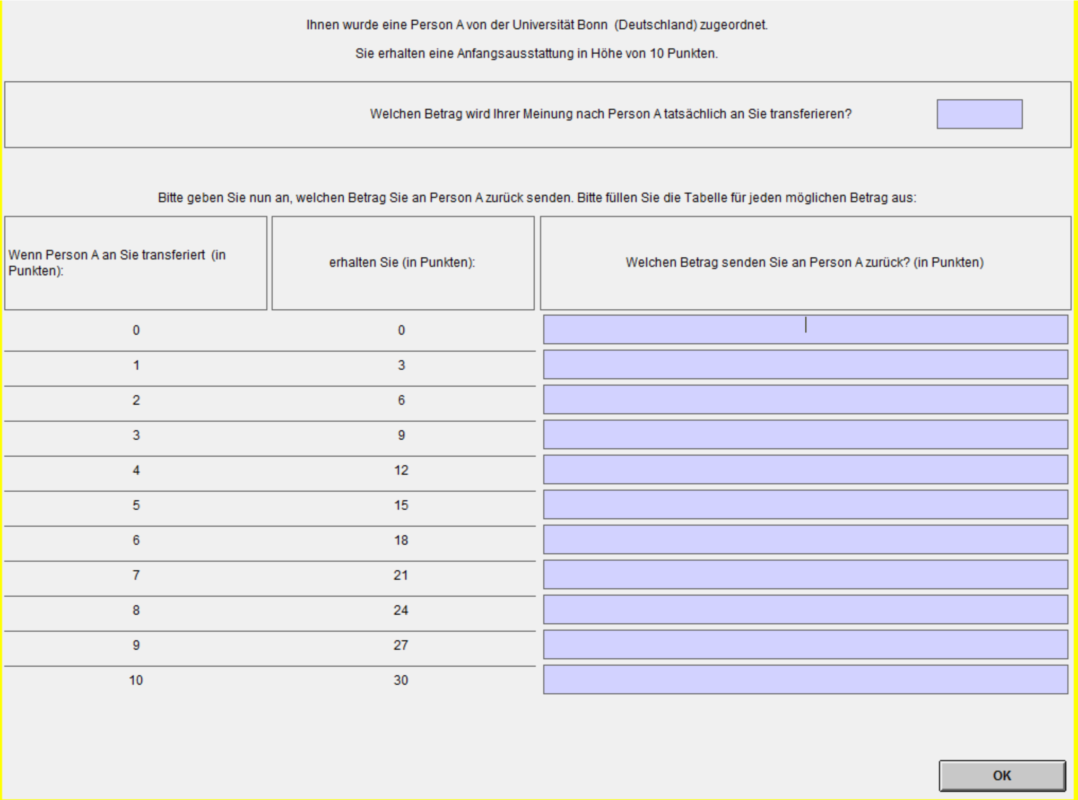

Ihnen wurde eine Person A von der Universität Bonn (Deutschland) zugeordnet.
Sie erhalten eine Anfangsausstattung in Höhe von 10 Punkten.

Welchen Betrag wird Ihrer Meinung nach Person A tatsächlich an Sie transferieren?

Bitte geben Sie nun an, welchen Betrag Sie an Person A zurück senden. Bitte füllen Sie die Tabelle für jeden möglichen Betrag aus:

Wenn Person A an Sie transferiert (in Punkten):	erhalten Sie (in Punkten):	Welchen Betrag senden Sie an Person A zurück? (in Punkten)
0	0	
1	3	
2	6	
3	9	
4	12	
5	15	
6	18	
7	21	
8	24	
9	27	
10	30	

OK

Section D References

S1. Greiner B. Subject pool recruitment procedures: organizing experiments with ORSEE. Journal of the Economic Science Association 2015; 1:114–125. DOI 10.1007/s40881-015-0004-4.

S 2 . Fischbacher, U. z-Tree: Zurich toolbox for readymade economic experiments. Experimental Economics 2007; 10: 171-178.
